# Pharmacological Agents Used in the Prevention and Treatment of Actinic Keratosis: A Review

**DOI:** 10.3390/ijms24054989

**Published:** 2023-03-05

**Authors:** Domenico Arcuri, Brandon Ramchatesingh, François Lagacé, Lisa Iannattone, Elena Netchiporouk, Philippe Lefrançois, Ivan V. Litvinov

**Affiliations:** 1 Department of Medicine, McGill University, Montreal, QC H4A 3J1, Canada; 2 Division of Dermatology, McGill University Health Center, Montreal, QC H4A 3J1, Canada

**Keywords:** actinic keratosis, squamous cell carcinoma, SCC, NMSC, pharmacotherapy, 5-FU, acitretin, nicotinamide, calcipotriol, salicylic acid, imiquimod, diclofenac, photodynamic light therapy, PDT, daylight, dPDT, ALA, MAL

## Abstract

Actinic keratosis (AK) is among the most commonly diagnosed skin diseases with potentially life-threatening repercussions if left untreated. Usage of pharmacologic agents represents one of many therapeutic strategies that can be used to help manage these lesions. Ongoing research into these compounds continues to change our clinical understanding as to which agents most benefit particular patient populations. Indeed, factors such as past personal medical history, lesion location and tolerability of therapy only represent a few considerations that clinicians must account for when prescribing appropriate treatment. This review focuses on specific drugs used in either the prevention or treatment of AKs. Nicotinamide, acitretin and topical 5-fluorouracil (5-FU) continue to be used with fidelity in the chemoprevention of actinic keratosis, although some uncertainty persists in regard to which agents should be used in immunocompetent vs. immunodeficient/immunosuppressed patients. Topical 5-FU, including combination formulations with either calcipotriol or salicylic acid, as well as imiquimod, diclofenac and photodynamic light therapy are all accepted treatment strategies employed to target and eliminate AKs. Five percent of 5-FU is regarded as the most effective therapy in the condition, although the literature has conflictingly shown that lower concentrations of the drug might also be as effective. Topical diclofenac (3%) appears to be less efficacious than 5% 5-FU, 3.75–5% imiquimod and photodynamic light therapy despite its favorable side effect profile. Finally, traditional photodynamic light therapy, while painful, appears to be of higher efficacy in comparison to its more tolerable counterpart, daylight phototherapy.

## 1. Introduction

Actinic keratosis (AK), synonymously referred to as solar keratosis, is among the most commonly diagnosed skin pathologies by dermatologists in the United States and Canada [1]. AK lesions are frequently found on sun-exposed areas of the body, such as the face, neck, dorsum of the hands, forearms and lower legs [2]. The risk of developing AK appears to be associated with cumulative ultraviolet (UV) exposure, as older individuals with lighter phototypes (Fitzpatrick I or II) tend to be the most vulnerable to developing the disease [3,4]. Indeed, studies analyzing AK prevalence among various age groups have revealed higher rates of the disease in countries whose populations have greater solar UV exposure (Australia vs. the United Kingdom), with older demographics (>60 years) demonstrating elevated prevalence compared to younger individuals (<40) [5]. Randomized control trials (RCTs) have demonstrated a decrease in both the incidence and the development of additional AK lesions if using sunscreen [6]. Indeed, while the most effective strategy in preventing AK is avoidance of UV radiation (UVR), photoprotection such as sun-protective clothing and sunscreen use, serve as important mitigation strategies. Furthermore, individuals receiving common photosensitizing medications such as hydrochlorothiazide have long been suspected of being at higher risk for developing the disease [7].

In line with the common association between excessive UV exposure and the development of skin cancer, it is widely accepted that AK serves as the precursor to cutaneous squamous cell carcinoma (SCC) [8] and, if left untreated, has the potential to transform into such. This association is strengthened via genetic analysis of AK and SCC lesions, with both demonstrating signature UVB-mediated mutations in p53, among other genes [9]. UVB rays particularly interact with the basal layer of the interfollicular epidermis, damaging its DNA and catalyzing the formation of SCC [10]. UVA rays, while more abundant in the environment and deeper-penetrating into the skin (dermis), are not as intimately associated with keratinocytic dysplasia, instead promoting damage via the formation of indiscriminate free (hydroxyl) radicals [5]. Importantly, lapses in immune function (e.g., iatrogenic immunosuppression in solid organ transplant recipients (SOTRs)), known to be an important contributor to oncogenesis, were shown to promote SCC formation from AK precursors [11,12]. Interestingly, apart from UVR-mediated damage to key oncogenes, chronic sun exposure has also been shown to promote a state of epidermal immunosuppression [13]. 

**Figure 1:**
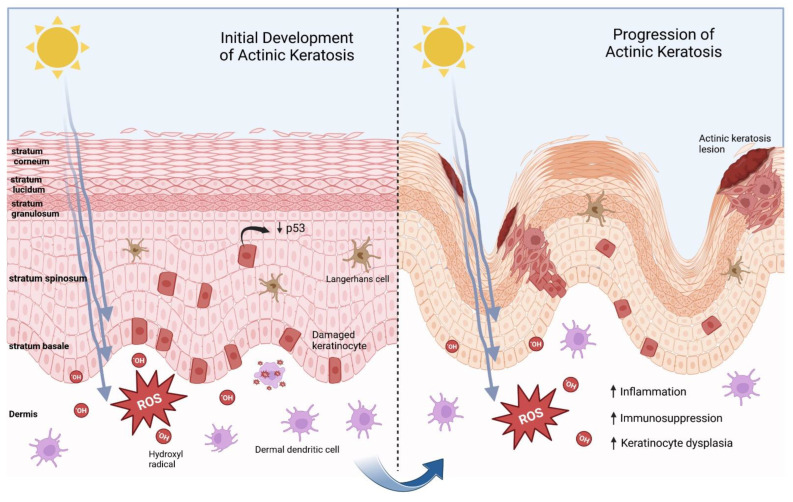
An overview regarding the initiation and progression of actinic keratosis lesions. The process is primarily driven by UVB-mediated keratinocytic DNA damage in the basal layer of the epidermis, particularly implicating tumor suppressor gene, p53. UVA-mediated reactive oxygen species (ROS) formation in the context of hydroxyl radical production in the dermis also promotes a pro-inflammatory, pro-tumorigenic environment via suppression of immune homeostasis and function. These factors combined allow for keratinocyte dysplasia to continue unchecked, eventually leading to the formation of visible actinic keratosis lesions on the surface of the epidermis.

AK lesions present along a spectrum of different phenotypes; they are usually characterized by scaly, erythematous, and sometimes hyperkeratotic papules that may be pruritic. The severity of an AK lesion is graded histologically, and is predominantly organized according to three degrees of keratinocytic atypia—keratinocytic intraepidermal neoplasia (KIN) I, KIN II or KIN III. KIN III—with the most significant of the grades referring to an AK that can also be considered as an SCC in situ [14]. Nevertheless, while AK biopsies can be used to prognosticate suspicious lesions, resultant grades do not habitually guide treatment options [15]. In fact, there are no clinical “gold standards” in classifying AKs, and treatment is often recommended regardless of the lesion’s morphological or histological underpinnings [16]. Figure 1 depicts a pathogenesis overview for actinic keratosis. 

Treatment selection is primarily guided by the overt features of AK, the size of the surface area being treated, demonstrated efficacy, tolerability of the enlisted intervention and patient preference [16]. Pharmacologic therapy in particular focuses on clearance and/or prevention of AK lesions, an approach that is derived from the theory of “field cancerization”. Field cancerization is the concept that areas of the body with extensive UV damage are prone to produce both higher numbers of AK lesions as well as lesions with greater potential to transform into SCC [17]. Cancerized skin may present with poikiloderma (hypo and hyperpigmentation telangiectasia and atrophy), or contain other features of dermatoheliosis and is important to treat [18,19]. Skin-directed pharmacologic therapy can be described as field-directed or lesion-specific (“spot treatment”). Field-directed therapies are capable of targeting larger areas of damaged skin and can therefore be used to both prevent and treat AK lesions [17]. Lesion-specific therapies are somewhat more destructive and are usually reserved for eradication of established AK lesions. Whether employing field-directed or lesion-targeted therapies, it is important to note that not all areas of the body are as amenable to treatment intervention. For example, it has been observed that AK presenting on the upper limbs is more difficult to treat compared to regions such as the face and scalp [20,21]. While many physical modalities can be used to treat AKs (cryotherapy, cauterization, curettage, and excision), this review will focus on pharmacologic interventions, analyzing research findings made over the past decade pertaining to drug efficacy, optimal dosage and adverse drug reactions (ADRs) in order to help both clinicians and patients select the most appropriate therapy. 

## 2. Chemoprophylaxis against AK

Chemoprophylaxis against AK can be defined as the prevention of (additional) AK lesions in the setting of UV-damaged skin. Unsurprisingly, there are strong recommendations and evidence favoring sunscreen usage in the prevention of AK lesions, mainly through the hinderance of initial and repeated UV-mediated damage [16,22]. While we acknowledge the essential role that sunscreen plays in the chemoprophylaxis against AKs, this section will focus on other commonly used pharmacologic chemoprotective interventions, namely oral/systemic nicotinamide, acitretin and topical 5-fluorouracil (5-FU). 

### 2.1. Nicotinamide Chemoprophylaxis

Nicotinamide, also known as niacinamide, the water-soluble amide form of vitamin B3, is involved in the formation of NAD+, a key intermediate in the generation of ATP. Interestingly, UVB-irradiated keratinocytes have demonstrated a marked decrease in NAD+, which is purported to increase tumorigenic potential secondary to loss of adequate energy production required for DNA repair [23]. Supplementation of keratinocytes with NAD+ has also been shown to increase DNA repair following UV-irradiation compared to placebo treatment [23]. In accordance with these results, it was hypothesized that increasing endogenous NAD+ levels, possibly through supplementation with nicotinamide, could lessen the formation of AKs and SCCs. Fortunately, nicotinamide has indeed been shown capable of enhancing repair of UV-mediated DNA damage in keratinocytes, reducing UV-mediated inflammation and protecting against UV-induced immunosuppression, all of which serve to limit the formation a pro-tumorigenic environment and solidify the drug’s potential as a favorable chemoprophylactic agent [24,25]. Clinical work in 2010 noted that 1% topical nicotinamide applied twice daily vs. vehicle alone reduced AK formation after 3 to 6 months of treatment [26]. This appears to be among the only published studies analyzing the topical formulation, as subsequent work has instead favored oral administration. A phase II dose-optimizing trial in 2012 determined that twice daily supplementation with nicotinamide 500 mg proved more effective at reducing AK counts with minimal reported ADRs compared to daily dosing [27]. 

It was a landmark phase III trial published a few years later, however, that substantiated nicotinamide 500 mg twice daily use as an effective and tolerable prevention strategy against AK lesions if used continuously for one year [28]. The strength of evidence associated with a 2022 meta-analysis citing a dose-dependent increase in digestive side effects (diarrhea) with nicotinamide use was considered to be very low [29]. Notably, most of the studies have been conducted in an immunocompetent population, although a number of publications regarding nicotinamide chemoprophylaxis in immunocompromised individuals also exist. A 2017 case–control study by Drago et al. did demonstrate a significant decrease in AK size in SOTRs taking 500 mg nicotinamide daily compared to placebo [30]. These results contradict findings made in 2016 by Chen et al.’s double-blinded phase II RCT, which noted non-significant decreases in AKs or keratinocyte carcinomas (KC)s in SOTR taking nicotinamide 500 mg twice daily [31]. Drago et al. suggested that this discrepancy may be due to a lack of consideration for the types and dosages of immunosuppressive drugs taken during Chen et al.’s study, subsequently affecting the efficacy of nicotinamide. Another small but notable difference is that while Chen et al. focused primarily on renal transplant patients, Drago et al.’s work also included liver transplant recipients (n = 8). A 2022 meta-analysis partially agreed with the findings made by Drago et al., resulting in a weak recommendation for the use of nicotinamide 500 mg twice daily in either immunocompetent or SOTR patients with a prior history of skin cancer [29]. Fortunately, a clinical trial assessing solo nicotinamide efficacy in immunocompromised individuals is currently underway and should better clarify the role that nicotinamide has in AK chemoprophylaxis for this population [32]. 

It has also been speculated whether or not nicotinamide could replace acitretin, a second-generation retinoid, for AK prophylaxis in SOTRs due to the former’s more favorable ADR profile. A 2021 meta-analysis revealed no significant efficacy difference between acitretin and nicotinamide in SOTR AK chemoprophylaxis, but could not comment on the duration of treatment, type of transplantation or optimal dosages of chemopreventative drugs [33]. Given the current lack of concrete findings supporting nicotinamide use in SOTR/immunocompromised individuals, it would be plausible to suggest utilizing nicotinamide in place of acitretin should the latter’s side effect profile prove intolerable to the patient. Another potential niche use for nicotinamide over acitretin would be due to the former’s lack of significant drug-drug interactions relative to acitretin, thereby reducing the risk of iatrogenic harm in a demographic traditionally known for polypharmacy [34]. Of note, there is little to no evidence either favoring or discouraging the simultaneous use of nicotinamide and acitretin in treating complex cases, although from a pharmacokinetic standpoint there appears to be little to no risk of significant drug interaction between either agent. 

### 2.2. Acitretin Chemoprophylaxis 

The most recent literature regarding acitretin chemoprophylaxis against AK has been focused on the SOTR population. Indeed, SOTR patients are at an increased risk of AKs and SCC due in part to a complex interplay between their chronically immunocompromised dispositions, exposure to potentially pro-carcinogenic medications and possible increases in susceptibility to UV radiation [35,36,37]. For decades, acitretin has been commonly used for the prevention of KCs in this demographic [38]. It works by binding all known subtypes of the retinoid X-receptors and retinoic acid receptors to normalize keratinocyte differentiation in the epidermis, as well as hindering the expression of pro-inflammatory cytokines such as IL-6, MRP-8 and IFN-γ [39]. Acitretin in doses measuring up to 30 mg per day has demonstrated beneficial effects on the number and thickness of AK lesions, as well as the number of new skin cancers [40]. A recent efficacy and cost analysis review of acitretin chemoprophylaxis in SCC and basal cell carcinoma (BCC) revealed a 54% and 73% reduction in both cancers, respectively, and suggested that acitretin may be underutilized due to its significant cost (in the United States), teratogenic risk and the need for regular blood test monitoring [41]. Nevertheless, acitretin has a notable mucocutaneous ADR profile which has been observed in individuals taking higher and subsequently more efficacious doses of the drug [40]. 

Patients on acitretin require monitoring for elevation in liver function tests and changes in triglyceride and lipid profiles. The drug should be avoided in females of childbearing age due to a significant risk of teratogenicity even up to 2–3 years after stopping the medication. This last point is important, as acitretin is metabolically converted to etretinate, a compound with a long-enough half-life to persist in the body for multiple years [42]. Studies have analyzed lower-dose acitretin regimens in an attempt to minimize ADRs while maintaining a similar efficacy. A 2022 retrospective case-crossover clinical trial tested 10 mg acitretin regimens in patients with one previous keratinocyte carcinoma and a history of SOTR and observed a 53% reduction in pretreatment KCs with no notable ADRs after at least two years of treatment [43]. Another retrospective cohort study published in 2022 analyzed long term (ranging from 6 months to 9 years) acitretin usage in SOTRs with a median/mode dose of 10 mg daily and found a 50% reduction in keratinocyte carcinomas during the first five years of treatment with only mild mucocutaneous ADRs [44]. In comparison to 30 mg doses, acitretin 10–20 mg daily does appear to maintain AK reduction capabilities in SOTR patients while reducing ADR incidence, although head-to-head trials are lacking. 

### 2.3. Topical 5-Fluorouracil Chemoprophylaxis

5-Fluorouracil (5-FU) is a thymidylate synthase inhibitor used in pathologies necessitating apoptosis of rapidly dividing cells. 5-FU is also known to increase p53 expression [45]. The Veterans Affairs Keratinocyte Carcinoma Chemoprevention (VAKCC) trial published in 2015 demonstrated that a single course of 5-FU 5% cream applied twice daily for up to 4 weeks on the face and ears decreased the incidence of new AKs for over two years [46]. Additional studies using data from the VAKCC trial surmised a 75% risk reduction in SCC, as well as a reduction in the need for surgical interventions caused by an SCC 1 year after treatment [47]. Furthermore, topical 5-FU usage has also been shown to incur lower costs over 3 years (USD 771) in comparison to placebo, making it a tolerable and cost-effective treatment strategy [48]. Common side effects of topical 5-FU include pain, pruritis, erythema and crusting, with rarer adverse events including infection and ulceration [49,50]. A post hoc treatment analysis revealed that twice daily 5% 5-FU led to a significant increase in the rate of crusting, scaling, erythema, stinging, burning and severe pruritis compared to patients taking 4% 5-FU daily [51]. Reducing the dosing frequency of 5-FU (weekly vs. daily) effectively diminishes the incidence of ADRs but comes at the cost of treatment efficacy and is generally not supported in clinical practice [52]. Unfortunately, the dose-dependent nature of 5-FU ADR onset entails that effective treatment with the drug is invariably intertwined with unpleasant side effects. Nevertheless, 5-FU, alongside nicotinamide and acitretin, are viable and generally tolerable chemoprophylactic options for dermatologists seeking to impede AK development and progression. Table 1 provides a brief summary of the discussed randomized-controlled, case–control trials and cohort studies since 2010. 

## 3. Treatment Modalities for AKs

Patients already presenting with numerous AKs or those at high risk for developing AKs and SCC may not only necessitate chemoprevention but actual treatment. Ingenol mebutate, a once very popular medication used to treat AK, has been discontinued in the United States, Canada and elsewhere around the world. As such, our review will only focus on specific and available pharmacotherapies. Resultantly, retinoids as a class of medications will also not be assessed despite their demonstrated efficacy in AK. Nevertheless, an in-depth review on retinoid utilization has recently been published by our team [53]. 

### 3.1. Topical 5-Flurouracil Treatment

5-FU has been used for many decades in the treatment of AKs. Identical to its chemoprophylactic regimen, 5-FU 5% cream is used twice daily over the course of 2–4-weeks as a field-treatment in order to eradicate AK lesions in immunocompetent patients [8,46]. 5% 5-FU has also been studied in immunocompromised individuals, with twice daily applications to the face of SOTR patients (three-week treatment duration) resulting in 79% AK clearance after 12 months of follow-up [54]. Provided 5-FU’s irritating nature, researchers have attempted to identify less caustic dosing regimens. Specifically, 0.5% 5-FU applied daily for 4–6 weeks has been shown to have the same efficacy as and better tolerability than the twice-daily 5% formulation in reducing the number of AK lesions from baseline [55]. Advocates for the 0.5% formulation have also suggested its use in elderly patients in order to diminish systemic 5-FU absorption while increasing compliance for those struggling to tolerate higher doses of the drug [56]. Even strengths as high as 4% 5-FU have been found to be as efficacious and better tolerated than 5% 5-FU cream when applied daily or twice daily for up to 4 weeks [57]. In spite of these findings, 5% 5-FU continues to be favored likely due to the abundance of empirical evidence available at this dosage, as well as its demonstrated efficacy in multi-treatment comparative and cost-effectiveness trials [58,59]. 

Of special importance to clinicians are the rare toxicities associated with topical 5-FU application in patients with dihydropyrimidine dehydrogenase (DPD) deficiency (DPDD). DPD is involved in the rate-limiting step in 5-FU metabolism; deficiencies in the enzyme subsequently lead to toxicity-inducing accumulation of the drug in the body. Severe lethargy, fever, mucositis, weight loss and neutropenia have been reported following application of 5-FU on patients with suspected DPDD [60,61]. It has been suggested that testing be performed for DPDD prior to systemic use of 5-FU to prevent toxicity, although this call has not been made for topical formulations of the drug [62]. Nevertheless, should a patient present with diagnosed DPDD, it would be best to resort to other non-DPD-associated AK treatments. It is also worth counselling patients using topical 5-FU to terminate treatment and return for clinical testing should, in a very rare event, they develop the aforementioned systemic symptoms.

Nonetheless, dose optimization is only one approach at reducing 5% 5-FU-associated ADRs and many studies within the past decade have focused on modifying 5-FU delivery to minimize the ADR incidence. Transfersomes, a group of specialized drug-delivering liposomes, have been studied for topical 5-FU administration with noted improvements in skin penetration, drug retention and irritation potential [63]. Pre-treatment barrier breakdown via microneedling seemingly potentiates the effects of 5-FU, leading to higher clearance rates for both 5% and 0.5% 5-FU compared to drug administration alone [64]. Additionally, a 2020 RCT assessed whether concomitant use of either petrolatum, clobetasol propionate or a controlled-release skin barrier emulsion (CRSBE) decreased ADRs associated with 5-FU facial usage [65]. Petrolatum was shown to be the most effective intervention at reducing erythema and increasing hydration without affecting 5-FU efficacy. Treatment with a 70% glycolic acid and 5% 5-FU solution every 2 weeks has also been shown to be as effective and tolerable as twice daily application of 5% 5-FU cream alone [66]. New drug options and combination treatments are also actively being investigated. A presently ongoing phase I clinical trial is seeking to determine whether a novel plant-derived compound named GZ17-6.02, when used in conjunction with 5-FU, can better eliminate AK lesions [67]. Current in vitro results demonstrate higher eradication of AK cells compared to 5-FU, and researchers hope that concomitant administration of both compounds can be used as a future treatment option. 

#### 3.1.1. 5-Fluorouracil and Calcipotriol

There already exist a few well-established treatment strategies incorporating 5-FU. One widely used and significantly efficacious combination therapy is 5-FU with calcipotriol. In addition to the anti-cancer effects associated with 5-FU, calcipotriol is thought to induce a T-cell-mediated anti-tumorigenic response when applied topically [68]. 5-FU and calcipotriol has also been shown to increase HLA Class II and thymic stromal lymphopoietin (TSLP) expression in lesional keratinocytes, indicating immune-mediated rejection of the AK [68]. This effect is thought to be aided synergistically by 5-FU, as it has been shown that monotherapy with calcipotriol results in only a modest clearance after 12 weeks without evidence of a robust immune response [69]. 

A double-blinded, 2017 RCT sought to clarify the efficacy and safety of 0.005% calcipotriol and 5% 5-FU cream, applied twice daily for 4 days [68]. There was an 87% reduction in AK lesions on the face, ~76% reduction on the scalp, and ~69–79% reduction on the upper extremities by week 8. Additionally, 27% of patients experienced complete clearance of AK lesions on the face, with 82% of patients finding the combination to be more efficacious than previous treatments they had received. The study also mentions that one included immunosuppressed patient had lower actinic clearance after receiving 5-FU + calcipotriol, demonstrating the importance of an intact immune system in generating a response with this treatment combination. Burning and erythema were the most common ADRs noted, and a follow-up, blinded, prospective cohort study noted that erythema extent and intensity was much higher on the face and scalp than on either upper extremity 1 day following treatment, which they attributed to an intense CD4^+^ T-cell response [70]. Perilesional skin biopsies taken during this second study demonstrated a marked increase in epidermal CD4^+^ memory T-cells in 5-FU + calcipotriol-treated skin, indicating a form of immunologic memory that was found to help reduce the 3-year risk of SCC on the face and scalp. 

A 2021 retrospective chart review of data collected between 2016 and 2018 tested the combination of cryotherapy followed by no drug, 1% 5-FU, 1% 5-FU + 0.005% calcipotriol and calcipotriol alone [71]. Treatments were applied in 3-week cycles for 5 nights on the face and 7 days elsewhere, followed by a two-week rest period before recommencing treatment. Cryotherapy and 5-FU + calcipotriol demonstrated earlier and significant AK reduction compared to 5-FU or calcipotriol alone. These results also contribute to the hypothesis that 5-FU + calcipotriol act synergistically to induce hastened anti-tumorigenic effects. Curiously, patients reported less irritation using 5-FU + calcipotriol than 5-FU alone; the most commonly reported adverse events included transient redness, dryness and itching. Some practitioners have anecdotally chosen to administer cyclic 5-FU + calcipotriol until the skin is clear; however, no evidence supports this practice. 

#### 3.1.2. 5-Fluorouracil and Salicylic Acid

5-FU and salicylic acid (SA) is another efficacious combination treatment for AKs. SA is a keratolytic agent which causes physical destruction of keratotic lesions, including more difficult to treat hypertrophic AKs [72]. A pilot study published in 2010 using 0.5% 5-FU in combination with salicylic acid (SA) 10% observed complete clearance in 77% of assessed AK lesions with tolerable burning following four weeks of three times per week applications [73]. Indeed, many patients respond to treatment periods less than 6 weeks, although longer therapy durations may be required depending on lesion location (for example, forearms) or if the patient has a previous treatment history [74]. Field-directed treatment using 0.5% 5-FU + 10% SA for 12 weeks has significant complete- and partial-clearance of AK lesions with appreciable efficacy against notably hyperkeratotic lesions on the face and scalp [75]. A 2017 study also demonstrated efficacy for AK lesions located on the hands and forearms irrespective of cornification or hypertrophy severity [76]. Most recently, an observational study assessing the early response of 5-FU + SA in treating actinic keratosis through the use of the AKASI system, a newly proposed quantitative tool to assess AK severity on the head, noted 84% clearance of AK lesions after 12 weeks of follow-up [77,78]. It appears that 5-FU + SA is an effective treatment for AK lesions, and especially hyperkeratotic AKs. The notable erosions observed in these studies may either be directly due to SA-induced keratolysis or the increased ability of 5-FU to penetrate deeper into the skin following concomitant administration with SA. Regardless of the mechanism, the combination may be better suited as a spot treatment versus field-directed therapy due to this particular ADR. It would be of interest to assess how head-to-head trials including 5-FU + SA, 5FU + calcipotriol and 5FU alone would compare in treating hyperkeratotic lesions, lesions on the upper extremities, face and scalp. 

### 3.2. Imiquimod 

Imiquimod topical cream is an established treatment of AKs with original case reports dating back to 2001 [79]. Imiquimod stimulates cell-mediated attack against AK lesions via the production of IFN-α, TNF-α, IL-6 and IL-8 as well as through the recruitment of activated CD4^+^, CD8^+^ T cells and mast cells [79,80,81,82,83,84]. Our understanding is that imiquimod exerts this function through Toll-like Receptors 7 and 8 on antigen-presenting cells, while also simultaneously inducing cytochrome-mediated apoptosis [85,86,87,88,89]. Dosing of imiquimod has traditionally included an application of a 5% cream 3 times weekly for 12–16 weeks [82,83,90,91,92]. Shorter treatment durations (4 weeks) have demonstrated similar efficacy to 16-week trials [93,94]. Application of imiquimod more than 3 times per week is associated with a higher rate of systemic adverse events and decreased tolerability [95]. The 5% formulation is also effective for treating AK lesions in SOTR patients with no evidence of undesirable immunoactivation [96,97]. Recent studies have assessed the use of 3.75% imiquimod cream with satisfactory results; a 2014 trial demonstrated a reduction of approximately 18 AK lesions per patient 8 weeks after of imiquimod 3.75% use on the face and scalp [98]. A 2020 study analyzing two case reports found that using 3.75% imiquimod applied in a field-directed manner successfully treated early-stage AK lesions with only mild erythema, burning and fatigue [99]. Another small 2020 study using 3.75% imiquimod also found efficacious and safe outcomes in 13 immunosuppressed patients [100]. Nevertheless, the ability of either 5% or 3.75% imiquimod to trigger autoimmune-induced organ rejection in SOTRs needs to be carefully considered and more robust data on medication safety is needed before definitive recommendations can be made. Kidney transplant recipients, where dialysis is readily available should the rejection occur, appears to be the safest SOTR demographic. 

Adverse events for imiquimod 3.75% and 5% creams are typically comprised of local skin reactions such as erythema, pain, inflammation, erosion, scabbing and pigmentation which typically self-resolve following termination of treatment and are positive indicators of dermal immune activation. [82,83,90,91,92,93,95,101,102]. However, systemic, flu-like symptoms such as fatigue, myalgia, fever and headache have also been associated with imiquimod 5% use [95,103,104,105]. While cutaneous reactions appear to precede flu-like symptoms by 7–11 days, there is no evidence of systemic cytokine activation nor of an association between the severity of skin reactions and the onset of systemic symptoms [103,106]. Nevertheless, imiquimod has very little circulatory absorption and exerts its effects locally, which may explain why only few patients sporadically experience systemic ADRs [103]. 

### 3.3. Diclofenac as a Well-Tolerated but Only Mildly Effective Treatment Option

Diclofenac is a non-steroidal anti-inflammatory drug capable of inhibiting both cyclooxygenase-1 (COX-1) and cyclooxygenase-2 (COX-2), thereby preventing the formation of inflammatory mediators such as prostaglandins and thromboxanes. Traditionally, its mechanism of action in treating AK was thought to primarily involve reduction in angiogenesis and cell proliferation, as well as induction of apoptosis when administered in hyaluronic acid [107,108]. Recently, a 2019 study observed increased infiltration of dermal CD8^+^ T cells accompanied with high IFN-γ mRNA expression in diclofenac-treated AK lesions, suggesting an immune-mediated component in the drug’s mechanism of action [109]. Regardless, diclofenac’s suspected efficacy in treating AK lesions stems as far back as the late 1990s [110]. It has been studied at a dose of ~3% diclofenac in 2.5% hyaluronan (HA) gel applied twice daily for 30–90 days with favorable effect [111,112]. However, immunohistochemical and histopathologic assessments have revealed that a 12-week treatment period may not be sufficient to fully eliminate AK lesions [113]. Nevertheless, a multi-center, randomized, open label study compared a 3- vs. 6-month treatment course of diclofenac 3%/HA 2.5% while analyzing clinical and histopathologic clearance and determined no significant differences in treatment outcomes [114]. There is an otherwise dearth of studies seeking to optimize diclofenac dose or reduce the incidence of adverse events. Indeed, adverse events associated with diclofenac 3%/HA 2.5% are minimal and usually include well-tolerated erythema, pruritis and dryness [107]. A 2019 study combined diclofenac with a group of naturally occurring compounds suspected of having activity against AKs by using self-assembling nanoparticles to deliver the drug in vitro into pig ear skin [115]. While much work remains to be done, one combination coined “Hybrid 1” (a combination of diclofenac with the antiproliferative and antioxidant compound “HT” in a Nano3Hybrid20 formulation) proved promising to the researchers, who claimed that it could serve as a scaffold for the development of new AK and SCC treatments. Nevertheless, this compound remains to be tested in clinical studies. 

### 3.4. Tirbanibulin 

Tirbanibulin is a novel drug option in the treatment of AKs. It serves as a microtubule and Src kinase inhibitor, which elicits the drug’s antiproliferative effects, as well as a p53 inducer, thereby potentiating apoptosis in target cells [116]. Although it’s potential as an anti-tumorigenic agent has been discussed over a decade ago, its efficacy in AK treatment has only recently been established [117,118]. Phase I and II studies have utilized tirbanibulin 1% ointment over 25 or 100 cm^2^ once daily for 3–5 days during a 45 (Phase I) or 57 (Phase II) day evaluation period [116]. Complete clearance of AK lesions on the face and scalp were noted to be 43% (5-day course) and 37% (3-day course) at day 57. The sustained treatment response 12 months after day 57 measurements was observed to be 43% for the 5-day regimen versus 30% for the 3-day regimen. A phase III study divided 702 patients into two trials and observed complete clearance of AK lesions in 44% (Trial 1) and 54% (Trial 2) of patients at 57 days post-treatment [119]. However, the authors did note the recurrence of lesions in 47% of patients who initially presented with complete clearance one year following treatment termination and subsequently called for comparative trials between tirbanibulin and standard AK treatments (e.g., topical 5-FU) to better qualify the compound’s role in AK management. Encouragingly, the adverse event profile for tirbanibulin appears favorable, with transient erythema, flaking and scaling being most commonly reported [119]. Evidently, more studies are required to determine whether or not it can supersede 5-FU or imiquimod as a popular and reliable treatment option. 

### 3.5. Traditional Photodynamic Light Therapy

Photodynamic light therapy (PDT) in conjugation with 5-aminolevulinic acid (ALA) for the treatment of AK was initially described in the mid 1990s with promising success [120]. Similar trials published a few years later using the photosensitizer methyl 5-aminolevulinate (MAL) also yielded satisfactory results [121]. Indeed, PDT has remained an important treatment strategy for AKs; it relies on the higher uptake of ALA and MAL, two prodrugs which are eventually metabolized into protoporphyrin IX (PPIX), by neoplastic cells [122,123,124,125]. Subsequent illumination at particular wavelengths promotes PPIX-induced, mitochondrialy-mediated destruction of abnormal tissue relative to healthy skin [126]. A 2004 phase III trial determined that ALA 20% solution applied and dried onto the skin 14–18 h before blue-light exposure, believed at the time to be more potent than red-light exposure, led to complete AK clearance in 73% of patients after 12 weeks [127]. This particular regimen was also effective as a spot treatment against AKs on upper extremities, initially offering clinicians another viable alternative against these treatment-resistant lesions [128]. Nevertheless, innovations in ALA-delivery have demonstrated the clinical utility of red-light PDT. BF-200 ALA, a nanoemulsion gel formulation containing the equivalent of 10% ALA-HCl with better penetration into the epidermis, has shown 91% complete clearance of AK lesions located on the face and scalp 12 weeks after redlight exposure [129,130]. Later studies have also found success with red-light BF-200 ALA-PDT in clearing AK lesions located on the hands and arms [131]. Occlusion with ALA-impregnated patches is another alternative yet effective drug delivery method recently shown capable of treating AK lesions on the hands and arms [132]. Interestingly, a 2019 chart review including 59 patients determined that 10% ALA generally has similar efficacy and adverse effect profiles as 20% ALA with lower associated cost of treatment, further antiquating 20% ALA-PDT as a go-to therapy [133]. Red-light exposure has also been tested with regimens involving 16% MAL-PDT, producing an apparent 89–91% clearance rate at three months follow-up [134,135]. 

Recent efforts have explored new compounds and adjuvant strategies in an attempt to augment PDT efficacy. Low-irradiance PDT combined with erbium:YAG laser pre-treatment prior to 16% MAL application has shown benefit in the SOTR population compared to MAL-PDT alone [136]. TAPP, a novel porphyrin derivative, shows potential for AK and adnexal neoplasm therapy, although much work remains to be done [137]. Oral vitamin D3 supplementation at 10,000 IU daily for 5 or 14 days prior to debridement and followed by ALA 20% blue-light PDT significantly improved clinical responses with acceptable tolerability for lesions on the scalp and face after 3 and 6 months [138]. A 2020 study assessing microneedling prior to ALA-PDT found marginal improvements in efficacy with no apparent increases in painfulness [139]. Indeed, dermarolling, microneedling and elongated particles have all demonstrated increased penetration and retention of MAL, although further studies on the clinical implications, such as efficacy, pain and compliance are needed [140]. 

Pain, erythema and post-treatment inflammation tend to be the most common and significant side effects associated with PDT, although case reports with rarer events including anaphylaxis and erosive pustular dermatosis have also been documented [141,142,143]. Of the mentioned side effects, pain in particular represents a notable adverse event which has limited the widespread use of PDT [144]. Interestingly, ALA is more commonly implicated with pain compared to treatments using MAL as a photosensitizer [145]. 

Strategies such as simultaneous application of 20% ALA with immediate blue-light irradiation have been shown to reduce pain compared to conventional application of ALA hours before exposure to a light source [146]. Simply shortening the drug-to-light period (for example, 1.5 hours vs. 3 hours) and allowing for 2 minute pauses during red-light illumination with ALA has also been shown to increase procedural tolerability [147]. Application with a topical anesthetic such as 7% lidocaine/7% tetracaine cream 1 h before MAL application is another useful strategy shown to significantly reduce PDT-associated pain [148]. A 2022 study found that substitution of the hydrochloride salt in ALA-HCl for phosphate (ALA-P) did not appear to improve treatment efficacy, but did lower perceived pain and favored absorption compared to either ALA-HCl or MAL-HCl [149]. Occlusion of BF-200 ALA on the scalp and face prior to illumination has been associated with increased pain, although efficacy notably improved as well [150]. Given the acceptable efficacy of PDT and already established association with patient reported pain, it is unlikely that occlusion of AK lesions on the scalp and face will be widely adopted into practice. Textile PDT using the FLUXIMEDICARE device, which advantages light-emitting knitted fabrics adaptable to the area of skin being treated, has been shown to be efficacious, tolerable and minimally painful [151,152]. 

#### Daylight Photodynamic Therapy

Daylight PDT (dPDT) is considered to be less painful than the traditional PDT [153]. The protocol involves exposure to a natural light following application of a photosensitizer, allowing dermatologists to treat AKs without the necessary irradiation equipment. A 2019 Phase III trial using BF-200 ALA demonstrated tolerability and non-inferiority compared to MAL-dPDT in the clearance of AK lesions 12 weeks following the treatment, indicating both compounds are viable photosensitizers for daylight therapy [154]. Artificial white light alternatives have been explored due to the variability in weather conditions and, therefore, unpredictability associated with dPDT administration. Devices such as Dermaris, which deliver uniform illumination of white light, have been shown to be effective and nearly painless treatment options for patients with AK lesions on the scalp [155]. Follow-up studies using the Dermaris device noted equal efficacy when diminishing the illumination period from 2.5 h to 1 h, all the while maintaining a nearly painless treatment of AK lesions on the scalp [156]. Another artificial dPDT device, IndoorLux, also demonstrated notable efficacy and relative painlessness following a treatment [157]. Needless to say, artificial white light regimens require in-house devices and do not advantage environmental UV exposure. Pre-treatment methods including microneedling and CO_2_ laser use prior to dPDT also demonstrated better clinical and histological results compared to dPDT alone, indicating a possible role for physical interventions in dPDT as well as traditional PDT [158]. Sequential treatment with calcipotriol for 14 days followed by application of MAL-dPDT has recently been shown to be more effective in treating thicker, upper-extremity AK lesions compared to MAL-dPDT alone [159]. dPDT has also shown promise in the SOTR population, although further studies in this demographic are required [160]. 

### 3.6. Trial Comparisons of 5-FU, Imiquimod, Photodynamic Therapy and Diclofenac Treatment Options

A multitude of comparative studies exist between 5-FU and other AK treatment agents. The SPOT trial published in 2022 found that 5-FU 5% was more effective than imiquimod and sunscreen at both treating and preventing AK lesions in SOTRs [161]. The authors of this trial hypothesized that imiquimod was not fully capable of exerting its effects due to patient’s immunocompromised states. A cohort study published in 2018 also found that 5-FU was more effective in the short term (2 years) but not long term (5 years, equal effect) at preventing AK lesions compared to imiquimod [162]. A cost-effectiveness RCT conducted in 2020 also determined that 5-FU was both more effective, as well as less expensive, than imiquimod and MAL-PDT at treating AKs on the head and neck area after 12 months [58,59]. A recent comparison between imiquimod 3.75% and MAL-PDT demonstrated slightly higher AK clearance when using imiquimod (68.1% vs. 56.5%), with the author’s suggesting the potential for combination or sequential treatment with both modalities [163]. This contrasts findings of a 2007 RCT comparing 5-FU to imiquimod demonstrating greater imiquimod-induced clearance in immunocompetent patients [164]. 5-FU 5% has demonstrated greater efficacy but decreased tolerability after 8 weeks of treatment when compared to diclofenac 3% [165]. A 3-year comparative trial between imiquimod 5% and diclofenac 3% published in 2020 also found diclofenac to be inferior to imiquimod in clearing and preventing AK lesions [166]. A 2021 comparison between combination PDT (dPDT followed by conventional PDT) to conventional PDT alone found similar efficacy between both regimens and higher tolerability with combination PDT, noting mild local skin reactions as the only significant adverse event [167]. No comparative trials have been conducted between different formulations of 5-FU. Table 2 provides a brief summary of randomized-controlled, case–control and cohort treatment trials published since 2010. 

## 4. Conclusions

There exists a variety of different pharmacologic options used to prevent and treat AKs. The surveyed studies vary on their follow-up times, parameters measuring efficacy, patient population and areas of the body targeted for treatment. General patterns have emerged, with 5-FU being a relatively efficacious chemoprophylactic and interventional treatment option for patients with established or emerging AKs. Medications such as diclofenac, which are inferior to 5-FU, offer the benefit of a tolerable adverse event profile. Novel treatment options such as tirbanibulin may be promising, and alterations in drug delivery methods can improve efficacy of existing drugs while limiting adverse reactions. Photodynamic light therapy, despite being reputed as a painful and cumbersome intervention, has acceptable efficacy. It would be of interest to assess future studies directly comparing various medications while controlling for patient demographic, lesion location and efficacy parameters. Indeed, there are many more important studies to be performed before we can truly understand which drug regimens are optimal for the spectrum of patients presenting with AKs.

## Figures and Tables

**Table 1:** Summary of randomized-controlled, case–control and cohort chemoprophylactic intervention studies conducted since 2010.

Study (n = x) [Citation]	Field, Spot or Systemic	Location	Strength	Dosage	Results	ADR
Nicotinamide (Immunocompetent)						
Moloney et al., 2010 [26]	Field	Face, scalp, upper limbs	1% gel	Twice Daily	Adult: 21.8% count reduction—3 months 24.6% count reduction—6 months Elderly: 22% count reduction—3 months 10% count reduction—6 months (non-significant)	Not reported
Surjana et al., 2012 n = 36 (Study 1) n = 41 (Study 2) [27]	Systemic	Face, scalp, upper limbs	500 mg	Twice daily × 4 months (Study 1) Daily × 4 months (Study 2)	Study 1: 35% count reduction—2 months 35% count reduction—4 months Study 2: 15% count reduction—2 months 29% count reduction—4 months	Not reported
Chen et al., 2015 n = 386 [28]	Systemic	Face, scalp, forearms, hands	500 mg	Twice daily × 12 months	12 months: 23% less NMSC vs. placebo 13% count reduction vs. placebo	Not significant
Nicotinamide (Immunocompromised)						
Drago et al., 2017 n= 38 [30]	Systemic	Entire body	500 mg	Daily × 6 months	6 mo: Decreased AK size in 88% of patients 42% with complete clinical regression 0% of patients developed new AKs	Diarrhea × 1 case
Chen et al., 2016 n = 22 [31]	Systemic	Face, scalp, forearms, hands	500 mg	Twice daily × 6 months	6 months: 16% reduction in AK count (non-significant)	Not significant
Acitretin (Immunocompromised)						
Solomon-Cohen et al., 2022 n = 34 [43]	Systemic	Unspecified	10 mg	Daily × 2 years	≥2 years: 53% reduction in pre-treatment KC	Not significant
Allnutt et al., 2022 n = 101 [44]	Systemic	Unspecified	8.10–22.5 mg Mode: 10 mg	Daily × 2–9 years	Reduced KC development by at least 50% during first 5 years of treatment (IRR < 0.5)	Dose-dependant mucocutaneous xerosis, peripheral sensory neuropathy, visual hallucinations, diarrhea
5-Fluorouracil (Immunocompetent)						
Pomerantz et al., 2015 n = 932 [46]	Field	Face and ears	5% cream	Twice daily × 4 weeks	6 months follow ups: Fewer AKs compared to placebo (3.0 vs. 8.1) at 6 months and duration of the study Higher AK clearance vs. placebo (38% vs. 17%) Fewer hypertrophic AK lesions (0.23 vs. 0.41)	Erythema, pruritus, burning, soreness, tenderness, crusting, erosions, scaling, flaking, and swelling
Weinstock et al., 2018 n = 932 [47]	Field	Face and ears	5% cream	Twice daily × 4 weeks	12 months: 75% risk reduction in SCC (1% vs. 4% development)	See [44]. Notably, 87% of patients would repeat treatment course if effective in reducing future risk of skin cancer

**Table 2:** Summary of discussed randomized-controlled, case–control and cohort treatment interventions published since 2010.

Study (n = x) [Citation]	Field, Spot or Systemic	Location	Strength	Dosage	Results	ADR
5-Flurouracil (Immunocompetent)						
Jansen et al., 2019 n = 624 [58]	Field	Head	5% cream	Twice daily × 4 weeks	12 months: No treatment failure (>75% reduction) in 74.7% of 5-FU patients compared to 53.9% imiquimod, 37.7% MAL-PDT and 28.9% ingenol mebutate	Erythema, swelling, erosion, crusts, vesicles, scaling, pruritis, pain, burning
Kishi et al., 2018 n = 1 [60]	Field	Bilateral forearms	0.5% cream	Daily × 30 days	Discontinuation of medication with ongoing effect requiring hospitalization. DPDD suspected but not confirmed	Lethargy, fever, fatigue, fever, mouth erosions, painful mucositis, weight loss
Cohen 2018 n = 1 [61]	Field	Face, lower lip	5% cream	Daily × 1 week, then twice daily × 2 weeks	Discontinuation of medications while treating neutropenia. Recommencement of medications save 5-FU did not cause neutropenia	Severe neutropenia
Khalil et al., 2022 n = 44 [64]	Field	Face	5% cream 0.5% anyhydrous serum	Intervention: 1.0 mm microneedling + 5% twice daily × 3 days + placebo × 12 days Or 0.5% twice daily × 3 days + placebo × 12 days Control: 5% cream twice daily × 15 days or 0.5% cream twice daily × 15 days	No statistical differences noted between microneedling + 3 days of treatment vs. 15 days of treatment alone at 3 mo (AK count 0.55 vs. 0.30 for drug alone) 5% 5-FU alone superior to 0.5% 5-FU alone. Microneedling + 5% FU superior to microneedling + 0.5% 5-FU in reducing AK lesions	Increased rate of erythema, crusting, exfoliation, scaling in 5-FU alone vs. microneedling + 5-FU. Slight but non-different ADR noted for 0.5% 5-FU and microneedling + 0.5% 5-FU
Maarouf et al., 2020 n = 30 [65]	Field	Face	5% cream	5-FU: Twice daily × 2 weeks Intervention: Applied to half of face twice daily × 2 weeks	98.1% resolution of AK count by week 4 Clobetasol propionate 0.5% best at decreasing transepithelial water loss (TEWL) (*p* = 0.034), petrolatum jelly best at improving hydration (*p* = 0.019) and erythema (*p* = 0.014), CRSBE improved TEWL (*p* = 0.17) and hydration (*p* = 0.19) but no effect on erythema (*p* = 0.257)	Erythema, burning and scabbing with 5-FU. No suspected ADR for other interventions
Heuser et al., 2020 n = 17 [66]	Field	Upper limbs	5-FU: 5% cream Glycolic acid: 70%	5-FU: Twice daily × 2 months Glycolic acid: Every 15 days followed by 5-FU% solution on skin for 12H × 2 months	Significant reduction of 75% and 85.71% in the mean number of AK lesions and of 74.5% and 85.71% in the size of lesions on the upper limbs of patients treated with glycolic acid = 5% 5-FU solution and 5% 5-FU cream (*p*-value ≤ 0.001) No statistical difference between either treatment	Some erythema, pruritis and pain. No statistical difference between either treatment
5-Flurouracil (Immunocomprimised)						
Ingham et al., 2014 n = 8 [54]	Spot	Face	5% cream	Twice daily × 3 week	63 and 0% complete clearance rates at 8 weeks and 12 months, respectively. 100% patients had partial clearance (>75%) at weed 8 and 71% at 12 months, respectively. Average patients had 15 AK at week 0, 1 at week 8 and 3 at 12 months. Mean AK clearance rate was 98% at week 8 and 79% at 12 months	Mostly mild erythema, pruritis and flaking or scaling
5-Fluorouracil + Calcipotriol (Immunocompetent)						
Cunningham et al., 2017 n = 131 [68]	Field	Face, scalp, upper extremities	5-FU: 5% cream Calcipotriol: 0.005%	Twice daily × 4 days	Calcipotriol plus 5-FU vs. Vaseline plus 5-FU × 4 days led to an 87.8% vs. 26.3% mean reduction in the number of actinic keratoses in participants (*p* < 0.0001)	5-FU + calcipotriol led to more skin redness, burning sensation and delated erythema resolution compared to 5-FU + vaseline. No difference in redness onset, pruritis and scaling
Rosenberg et al., 2019 n = 86 [70]	Field	Face, scalp	5-FU: 5% cream Calcipotriol: 0.005%	Twice daily × 4 days	5-FU + calcipotriol–induced tissue-resident memory T (Trm) cell formation on face and scalp is associated with more erythema (*p* < 0.01). More epidermal Trm cells persisted in the 5-FU + calcipotriol–treated face and scalp skin (*p* = 0.0028) More participants remained SCC-free more than 1500-days after 5-FU + calcipotriol treatment (*p* = 0.0765), and significantly fewer developed SCC on the treated face and scalp within 3 years	Notable focus on erythema
Moore et al., 2021 n = 175 [71]	Spot (cryotherapy pre-treatment) + Field	Face	5-FU: 5% cream Calcipotriol: 0.005%	Three week cycles of 5 nights on the face, 7 nights elsewhere then 2 weeks off before repeating	5-FU + calcipotriol showed AKreduction at 101 to 200 days (9.55; *p* = 0.002) and 201 to 300 days (14.70; *p* = 0.001) post follow up. Small difference in AK clearance between 5-FU + calcipotriol (14.70; *p* ¼ 0.001) and cyclic vitamin D (14.18; *p* ¼ 0.004) at 201 to 300 days (Figure 1). 5-FU + calcipotriol demonstrates greater and earlier AK reduction compared to cryotherapy alone (*p* = 0.008)	Redness, dryness and pruritis
5-Fluorouracil + salicylic acid (Immunocompetent)						
Schlaak et al., 2010 n = 15 [73]	Spot	Face, scalp	5-FU: 0.5% cream SA: 10%	3 times per week × 4 weeks	12 weeks: Complete response in 77%, partial response in 21% and non-response of 1 (2%) of surveyed AK lesions was achieved	Burning named as most notable. Redness, irritation, dryness and peeling also present
Szeimies et al., 2015 n = 1051 [74]	Spot	Face, head, arms, hands, legs, trunk	5-FU: 0.5% cream SA: 10%	Once daily on up to 10 lesions	Mean Ak count decreased by approximately 70% during the observation period. Mean size of AK decreased by approximately 80%. About 50% of surveyed patients were treated less than 6 weeks	Pain, erythema, burning, irritation, discoloration, scabbing and erosion
Stockfletch et al., 2017 n = 166 [75]	Field	Face, scalp	5-FU: 0.5% cream SA: 10%	Once daily × 12 weeks	8 weeks following treatment: Complete clearance was found to be 49.5% vs. 18.2% with vehicle alone (*p* = 0.0006) Partial clearance was found to be 69.5% vs. 34.6% with vehicle alone (*p* = 0.0001). 99.1% of assessed patients experienced adverse events with treatment	Erythema, inflammation, and scabbing
Reinhold et al., 2017 n = 649 [76]	Spot	Hands, forearms	5-FU: 0.5% cream SA: 10%	Once daily to a maximum of 10 lesions	8 weeks after end of treatment: AK count reduction by 92% (0.3 lesions per patient (*p* < 0.0001)) Decrease in the size of the lesions by87% (*p* < 0.0001)	Erosion, irritation, pain, discharge, erythema, bleeding, macula, pruritis, rash, scar, ulcer in only 2% of patients
Garofalo et al., 2022 n = 40 [77]	Field	Face, scalp	5-FU: 0.5% cream SA: 10%	Once daily for 12 weeks	AKASI score decreased from an initial score of 3.3 to a final score of 0.9. 12 week: 84% of assessed lesions showed complete clearance, partial clearance observed in 8%	Erythema, pruritis, erosion, bleeding
Imiquimod (Immunocompetent)						
Stockfletch et al., 2014 n = 319 [98]	Field	Face, scalp	3.75% cream	Daily × 2 weeks on, off, on	8 week after treatment: Median of 18 AK lesions werecleared corresponding to a median percentage reduction of 92.2% of all the patients’ AK lesions compared to 39.3% for placebo	Not reported
Kopera 2020 n = 2 [99]	Field	Face	3.75% cream	Twice daily × 2 weeks	Complete healing within 2–4 weeks of AK lesion without sequelae	Burning, fatigue, mild erythema
Imiquimod (Immunocompromised)						
Zavattaro et al., 2020 n = 13 [100]	Field	Scalp	3.75% cream	Daily × 2 week on, off, on	8 weeks follow up: Complete clearance in 46% of patients 38% of patients had a 50% reduction in AK count 15% of patients had an 80% reduction in AK count	Erythema, crust, rarely edema, asthenia and fatigue
Bhatia et al., 2022 n = 22 [106]	Field	Face, scalp, trunk, upper extremities	3.75% cream	Daily × 2 week on, off, on	Systemic symptos occurred rarely but usually followed local skin reactions within a 7–11 day period	Local skin reactions assayed included symptoms erythema and pruritis. Some assayed systemic symptoms included fever, headache and fatigue
Diclofenac (Immunocompetent)						
Singer et al., 2019 n = 28 [109]	Spot	Unspecified	Diclofenac: 3% Hyalorinic Acid: 2.5% gel	Twice daily × 12 weeks	Gene expression of *glucose transporter-1* (*GLUT-1*) was increased in AK lesions compared to normal skin Decrease in epidermal CD1a+ cells but increased dermal CD8+ T cells in AK Diclofenac treatment reduced AK lactate and amino acid levels while inducing infiltration of dermal CD8+ T cells and high *IFN-*γ mRNA expression	Not reported
Çayirli et al., 2013 n = 44 [113]	Spot	Face, scalp	Diclofenac: 3% Hyalorinic Acid: 2.5% gel	Twice daily × 12 weeks	Immunohistochemical and histopathologic examinations revealed that 12-weeks might not be enough to treat AK Ki-67 (*p* = 0.042) and p63 (*p* = 0.030) expression decreased denoting an anti-proliferative effect Complete clearance seen in 19 lesions (32.8%). Significant improvement seen in 25 lesions (43.1%) and mild-moderate improvement in 9 lesions (15.5%). No improvement in 5 lesions (8.6%), Complete remission was observed at a significantly higher rate in Grade 3 lesions (*p* = 0.017)	Xerosis, erythema, crusting
Pflugfelder et al., 2012 n = 418 [114]	Unclear	Face, head	Diclofenac: 3% Hyalorinic Acid: 2.5% gel	Group A: Twice daily × 3 months Group B: Twice daily × 6 months	Complete clearance in 40% (Group A) and in 45% (Group B) of AK lesions (*p* = 0.38). Histopathological clearance in 30% (group A) and 40% (group B) of AK lesions (*p* = 0.16). Decreased size in 38% (group A) and 39% of (group B) of surveyed AK lesions	Erythema, scaling, edema, erosion, induration
Tirbanibulin (Immunocompetent)						
Kempers et al., 2020 n = 30 (phase I) n = 168 (Phase II) [116]	Field	Phase I: Forearms Phase II: Face, scalp	1% ointment	Daily × 3 or 5 days	Phase I: By day 45, 25% (50 mg over 25 cm^2^ × 3 days), 0% (200 mg over 100 cm^2^ × 3 days), 50% (50 mg over 25 cm^2^ × 5 days), and 12.5% (200 mg × 5 days over 100 cm^2^) of participants demonstrated complete AK clearance Phase II: More participants had complete clearance at day 57 in the 5-day vs. the 3-day cohort (at 50 mg over 25 cm^2^) (43% vs. 32%) Partial clearance rates were higher in the 5-day vs. the 3-day cohort (at 50 mg over 25 cm^2^) (56% vs. 52%) An overall average decrease in AK count occurred by day 15 in the 5-day (−2.5 [2.48]) vs. 3-day (−2.5 [2.22]) regimens which continued up to day 57 (−3.9 [2.00] and −3.4 [1.75], respectively)	Erythema, scaling, crusting
Blauvet et al., 2021 n = 702 [119]	Field	Face and scalp	1% ointment	Daily × 5 days	Day 57: Complete AK clearance in 174 of 353 patients (49%) using tirbanibulin vs. vehicle (9%) after pooling data from both trials (44% clearance in Trial 1, 54% clearance in Trial 2). 12 mo: 47% AK recurrence in patients who initially had a complete response	Erythema, flaking, scaling, pain, pruritis
Traditional Photodynamic Therapy (Immunocompetent)						
Berman et al., 2020 n = 269 [128]	Spot	Face, scalp, upper extremities	20% ALA BLU-U illuminator	ALA applied twice prior to illumination; repeated × 1 if lesions noted after 8 weeks	12 weeks follow up post-baseline: Clearance was 80.6% (vs. 45.5% placebo; *p* < 0.0001) and the mean decrease in cumulative disease area was 82.4% (vs. 42.6% placebo; *p* <0.0001)	Edema, erythema, hyperpigmentation, hypopigmentation, scaling, dryness, stinging, burning, oozing, vesiculation, crusting
Reinhold et al., 2016 n = 94 [129]	Field	Face and scalp	BF-200 ALA BF-Rhodo-LED Lamp	1 session repeated × 1 if lesions still noted after 12 weeks	12 weeks following treatment: ALA complete clearance at 91% (vs. 22% placebo, *p* < 0·0001) and complete clearance rate at 94·3% (vs. 32·9% placebo, *p* < 0·0001) after a maximum of two PDTs	Pain at application site, erythema, pruritus, scab, exfoliation, oedema and vesicles
Ulrich et al., 2021 n = 50 [131]	Field	Neck, trunk, extremities	BF-200 ALA BF-RhodoLED lamp (Biofrontera	1 session Maximum of 2 session permitted	Complete clearance rates were 86.0% (vs. 32.9% for placebo; *p* < 0.0001) and patient complete clearance per patient’s side was 67.3% (vs. 12.2% for placebo, *p* < 0.0001). One-year overall lesion recurrence rate was 14.1% (vs. 27.4% placebo *p* = 0.0068) Patients were more satisfied with cosmetic outcome of ALA/PDT than vehicle/PDT	Pain, erythema, pruritis, edema, scab, exfoliation, vesicles
Bai-Habelski et al., 2022 n = 20 [132]	Field	Hands, arms	PD P 506 A patch Aktilite CL 128 or BF-RhodoLED illuminator	3–8 AK lesions covered by one patch and illuminated × 1 followed by 2nd session 2 weeks later	Complete clearance at 78.0%(95% CI: [64.6%, 87.3%]), and the by-participant clearance calculation was at 78.7% (95% CI of [67.0%, 90.3%])	Erythema, irritation, pain, burning, discomfort, pruritus, exfoliation, desquamation, scab, excision, vesicles, edema, inflammation, headache
Bullock et al., 2022 n = 58 [139]	Field	Face, scalp	ALA 20% Vit D 10,000 IU	1 session	3 to 6 months: Mean clearance rates were lower in patients with vitamin D deficiency(40.9% +/− 42%) than in patients with normal vitamin D levels (62.6% +/− 14.2%). Vitamin D supplementation significantly improved the overall AK lesion response (72.5% 6 13.6%)	Pain, erythema, warmth, exfoliation, tightness, scabbing, edema, blistering, erosions, hemorrhage, discharge, pigmentary changes
Urvashi et al., 2020 n = 23 [146]	Field	Face, scalp	ALA 20% Blu-U Illuminator	1 session	Less pain during simultaneously illumination compared to conventional PDT 3 months follow up showed nearly identical clearance with bothsimultaneous and conventional treatment asdetermined by statistical testing of noninferiority +/− 15% margin	Burning, itching, redness, stinging, swelling, crusting, peeling
Salvio et al., 2021 n = 30 [147]	Field	Forearms, hands	ALA 20% 630 nm LED Prototype	1 session	Pain comparison showed best results when illuminating 1.5 h with 2 min breaks compared to conventional PDT 30 days: No statistical significant difference in clearance when illuminating 1.5 h with 2 min pauses compared to conventional PDT	Pain predominantly assessed
Brumana et al., 2020 n = 50 [148]	Field	Face, scalp	MAL 16% 7% lidocaine/7% tetracaine cream Akilite Lamp	1 session	Median values of pain VAS score with anesthetic application was reduced by 60% vs. placebo (3.0 vs. 7.5) (*p* = 0.0009)	Pain predominantly assessed
Bartosińska et al., 2022 n = 22 [149]	Field	Face, scalp	ALA-HCl: 12.7% MAL-HCL: 12.5% ALA-P: 17.5% Red Beam Pro+	1 session	Pain intensity during PDT was significantly lower with ALA-P (5.8 on average) in comparison to the areas treated with ALA-HCl or MAL-HCl (7.0 on average on 0–10 scale) 94% of patients rated obtained cosmetic effect as excellent. No significant difference in efficacy	Erythema, edema, desquamation, crusting, and pustules
Meierhofer et al., 2020 n = 45 [150]	Field	Face, scalp	BF-200 ALA BF-RhodoLED	1 session	3 months: Clearance rate of the target AK and total AK after PDT was 88.4% and 90.6% with occlusion and 58.1% (*p* = 0.001) and 70.4% (*p* = 0.04) with non-occlusion. 6 months: Clearance of target and total AK was 69.7% and 72.1% with occlusion and 30.2% (*p* < 0.001) and 35.6% (*p* = 0.001) with non-occlusion. Pain score and skin phototoxicity were significantly higherafter occlusive ALA application	Photoxicity assessed as the sum of erythema, edema, blistering
Vicentini et al., 2019 n = 25 [151]	Field	Forehead, Scalp	MAL 16% FLUXIMEDICARE	1 session, then 1 session × 3 months later if AK still present	3 months: Clearance was non-inferior to that obtained with the conventional PDT (660% vs. 591%,respectively; absolute difference, 69%; 95% confidence interval–06% to 145%). Pain was significantly lower with the Flexitheralight protocol vs. conventional PDT (*p* < 00001)	Pain, erythema, edema
Dubois et al., 2021 n = 39 [152]	Field	Forehaead, scalp	MAL 16% FLUXIMEDICARE	1 session	3 months: Clearance was 72.6% (95% CI 67.9–77.0) 6 months: Clearance was 67.5% (95% CI 61.2–73.3)	Pain, erythema
Traditional Photodynamic Therapy (Immunosuppressed)						
Lonsdorf et al., 2022 n = 18 [136]	Field	Face, scalp	16% MAL BF-RhodoLED^®^	1 session	3 months: Low-irradiance photodynamic therapy combined with Er:YAG pre-treatment lesion re- sponse rate of 77.3 ± 23.6%) compared to MAL-PDT(61.8 ± 21.4%; *p* = 0.025) without worsening pain (*p* = 0.777) or cosmetic outcome (*p* = 0.157)	Pain
Daylight Photodynamic Therapy (Immunocompetent)						
Dirschka et al., 2019 n = 52 [154]	Field	Face, scalp	BF-200 ALA MAL 16%	1 session	12 weeks: Complete clearance for 79.8% of AK lesions treated with BF-200 ALA gel and 76.5% of the lesions treated with MAL (*p* < 0.0001). 12 months: Recurrence for 19.9% of lesions treated with BF-200 ALA and 31.6% for lesions treated with MAL	Erythema, pain, pruritus, scab
Maire et al., 2020 n = 38 [155]	Field	Scalp	MAL 16% Dermaris	1 session, repeated at 3 mo if more than 5 AK lesions present	3 months: Complete clearance for 58% of patients after the initial treatment. 32% required another round of PDT11% of patients showed 1–4 AK lesions remaining, all of which weregrade I–II and subsequently cured with topical ingenol mebutate. 87% of patients experienced no pain Discomfort, pruritus rated as mild or less (97%)	Pain, pruritus,, discomfort, crusting evaluated
Creusot et al., 2021 n = 30 [156]	Field	Scalp	MAL 16% Dermaris	1 session, repeated at 3 mo and 6 mo if lesions were still present	6 months: 93% clearance reported Twenty-six patients (87%) experienced no pain during the first PDT	Mild pain, erythema, crusting, discomfort
Bai-Habelski et al., 2021 n = 12 [157]	Field	Face, scalp	BF-200 ALA IndoorLux	2 session with no pre-defined interval between both treatments	Median clearance rate after second treatment was 83.75%33.3% of patients demonstrated complete clearance. Median size of the remaining lesions decreased by 42.9%. The first treatment was pain-free for 58.3%of patients	Pain predominantly assessed
Bento et al., 2021 n = 40 [158]	Field	Face	MAL 16%	2 sessions 4 weeks apart	dPDT + physical interventions had better clinical and histologic results. AK-clearancewas higher after both 1 and 3 months with pretreatment-CO_2_ laser	Pain, erythema, edema
Piaserico et al., 2021 n = 36 [159]	Field	Dorsum of hands, forearms	MAL 16% Calcitriol 3 mg/g	2 sessions 1 week apart Calcitriol: Daily before bedtime × 14 days	After 3 months, the overall lesionresponse rate and patient ≥ 75% clearance rate of CAL-DL-PDT were higher, albeit not significantly, than P-DL-PDT. According to grade, response rate of grouped AK II/III was significantly higher for CAL-DL-PDT than for P-DL-PDT while similar results were observed for grade I AK	Calcitriol: Erythema, itch dPDT: Erythema, edema, crusting, pustulation
Daylight Photodynamic Therapy (Immunodeficient)						
Bernard et al., 2020 n = 24 [160]	Field	Face, scalp	MAL 160 mg/g	2 sessions 15 days apart, followed by double sessions at 3 and 9 months	Daylight PDT showed significantly lower mean of new AK lesions compared to control side 3 months (4.2 [3.4] vs. 6.8 [4.8]; *p* < 0.001), 9 months (3.0 [3.3] vs. 4.3 [3.4]; *p* = 0.04) and 15 months (3.0 [4.6] vs. 4.8 [5.0]; *p* = 0.02) after treatment. Mean number was non-significant at 21 months (3.7 [3.5] vs. 5.0 [4.5]; *p* = 0.06). Most participants favored DPDT	Erythema, inflammation, blisters, crusting, pruritus, desquamation, burning, stinging
Comparative Trials (Immunocompetent)						
Neugebauer et al., 2018 n = 5700 [162]	Field	Unspecified	5-FU Imiquimod	Unspecifed	5-FU reduced the short-term incidence (cumulative risk difference -4.54%), but not long-term incidence (cumulative risk difference -1.43%) of AKS compared to imiquimod treatment	Unspecified
Cortelazzi et al., 2020 n = 9 [163]	Field	Scalp	Imiquimod 3.75% 16% MAL Aktilite Lamp	Imiquimod: Daily for 14 days on, off on 14 days after treatment with MAL-PDT MAL-PDT: 1 session	Imiquimod has higher overall clearance rate than MAL-PDT (68.1% vs. 56.5%) Higher clearance rates for I and III degree AKs with imiquimod (68.8%, 64.5% and 75%) vs. 48%, 69.8%, and 66.7% with MAL-PDT) A higher total recurrence rate was noted for imiquimod compared with MAL-PDT (9.9% vs. 8.6%) after 12 months	Both treatments: burning, erythema, edema, erosions, and crusts, flu-like symptoms (fever, asthenia, headache, joint pain)
Segatto et al., 2013 n = 28 [165]	Unclear	Face, scalp, hands	5-FU 5% Diclofenac 3% hyaluronic acid 2.5%	5-FU: Twice daily × 4 weeks Diclofenac/Hyaluronic acid: twice daily × 12 weeks	Significant reduction in the number of AK lesions with 5-FU vs. diclofenac (*p* < 0.001). High degree of satisfaction for both 5-FU vs. diclofenac (73% and 77%, respectively; *p* = 0.827)	Erythema, edema, crusts and itching were significantly higher with 5-FU
Gollnick et al., 2020 n = 479 [166]	Field	Face, scalp	5% Imiquimod 3% Diclofenac	Imiquimod: 3 nights per week × 4 week followed by 4 week treatment pause; additional 4 week treatment if lesions noted Diclofenac: Twice daily × 12 weeks	Grade III AK or invasive SCC transformation was observed until 3 yrs in 5.4% of patients treated with imiquimod vs. 11.0% of patients treated with diclofenac (absolute risk difference –5.6% [95% CI: 10.7%, –0.7%]) Time to histological change was longer with imiquimod vs. diclofenac (*p* = 0.0266)	Imiquimod: Pruritus, pain, irritation, inflammation, alopecia, anaemia, psoriasis Imiquimod: Pruritus, pain, dermatitis, irritation, inflammation, rash, alopecia, anemia
Sáenz-Guirado et al., 2022 n = 51 [167]	Field	Face, scalp, forehead	BF-200 ALA	Conventional PDT: 1 session ComboPDT: Daylight PDT followed by conventional PDT	Grade I and II AK reduction rate was similar between combo PDT and conventional PDT, with no statistically significant differences between either groups (Grade I: 76.67% vs. 86.63% [*p* = 0.094]) and (Grade II: 80.48% vs. 83.08% [*p* = 0.679]). Pain was significantly lower in the combo PDT group (2.56 vs. 5, *p* < 0.01), including local skin reactions	Combo PDT: Erythema, edema, crusting Conventional PDT: Erythema, Edema, Flaking, Crusting
Comparative Trials (Immunosuppressed)						
Hasan et al., 2022 n = 40 [161]	Field	Head, Neck, Upper Limb	5-FU Imiquimod 5%	As used in routine clinical practice, with repeat treatment permittable after 4 weeks	5-FU and imiquimod were superior to sunscreen for AK clearance and prevention. 5-FU in particular was also more effective than imiquimod in AK clearance and prevention	5-FU: Pruritus, fatigue, flu-like symptoms, headache, myalgia, photosensitivity, malaise, arthalgia, nausea, Imiquimod: Pruritus, fatigue, hypopigmentation, flu-like symptoms, headache, myalgia, dizziness, malise, arthalgia, nausea, vomiting, diarrhea, bruising

## Data Availability

All data is presented in the paper.
